# Informed consent in gynecological oncology: a JAGO/NOGGO survey on real-world practices in daily clinical routine

**DOI:** 10.1007/s00404-024-07776-9

**Published:** 2024-11-01

**Authors:** M. G. Biersack, L. L. Volmer, C. Geißler, J. Fromme, S. Fröhlich, K. Pietzner, J. Sehouli, M. H. Beck

**Affiliations:** 1 Department of Women’s Health, University of Tübingen, 72076 Tübingen, Germany; 2 Young Academy of Gynecologic Oncology (JAGO), Nord-Ostdeutsche Gesellschaft für Gynäkologische Onkologie, 13359 Berlin, Germany; 3 Department of Gynecology With Center for Oncological Surgery, Campus Virchow Klinikum, Charité-Universitätsmedizin Berlin, Corporate Member of Freie Universität Berlin and Humboldt Universität zu Berlin, Augustenburger Platz 1, 13353 Berlin, Germany; 4 Department of Women’s Health, Klinikum Fürstenfeldbruck, 82256 Fürstenfeldbruck, Germany; 5 Department of Women’s Health, University of Freiburg, 79106 Freiburg, Germany; 6 Department of Gynaecology and Obstetrics, St. Elisabeth-Krankenhaus, 50935 Cologne, Germany; 7 Department of Women’s Health, Klinikum Südstadt Rostock, University of Rostock, 18059 Rostock, Germany

**Keywords:** Informed consent, Gynecologic oncology, Gynecology, Survey, Patient education

## Abstract

**Purpose:**

Informed consent is a quintessential element of contemporary medicine, reflecting the fundamental right of patients to participate in decision-making regarding their health. Despite its critical importance, there is a lack of data on real-world practices regarding patient informed consent in the context of modern, high-pressure medical environments.

**Methods:**

We conducted a multinational multicentric survey from February 24, 2022, to September 14, 2022, investigating the practices and challenges surrounding informed consent in hospitals across Germany, Austria, and Switzerland with the use of a specifically developed questionnaire.

**Results:**

Drawing on over 200 responses from gynecologists, the survey shows a critical need for structured training in conducting informed consent discussions with over 80% of participants expressing interest in courses addressing this aspect. Notably, a considerable portion of the physicians (59.9%) reported conducting discussions on procedures they had never personally witnessed. Significant disparities between types of hospitals and professional groups were observed in the frequency of informed consent discussions, with limitations arising from factors such as time constraints, language barriers, and insufficient resources for patient education. Moreover, the psychological burden experienced by physicians after informed-consent discussions underscores the need for systemic changes to alleviate concerns regarding patient safety, legal repercussions, and patient satisfaction.

**Conclusion:**

This study serves as a call to action, emphasizing the need of enhancing resources and support for medical professionals to uphold the principles of empathic and comprehensive patient information and shared decision-making.

**Trial registry:**

DRKS00028295, 25.07.2024

**Supplementary Information:**

The online version contains supplementary material available at 10.1007/s00404-024-07776-9.

## What does this study add to the clinical work

**Table:** 

This multinational survey highlights the urgent need for structured training in conducting informed consent discussions. It serves as a call to action, underscoring the importance of enhancing resources and support for medical professionals to uphold the principles of empathy, comprehensive patient information, and shared decision-making.	

## Introduction

Since the time of Hippocrates, the benefits of medical care have been highly valued, but it was not until the nineteenth century that patient autonomy and involvement in decision-making began to gain recognition. Before this shift, the primary focus was often on pacifying rather than informing patients, to the extent that deceiving patients was widely accepted [1]. In the present day, every individual has the right to their own physical integrity. As a result, informed consent is necessary to legitimate any elective invasive medical or surgical procedure, for it not to be legally considered as battery [2]. The German Civil Code (§ 630c-e BGB) outlines the informed consent process, requiring healthcare providers to ensure that patients are informed comprehensively and in a timely manner about all relevant aspects of a planned procedure. Informing physicians must also possess the necessary training to perform the procedure.

The extent to which the patient should be informed is a controversial subject. Finding the right balance between presenting all possible risks and complications to meet the legal requirement while not causing unnecessary anxiety or insecurity in patients, can be challenging [3, 4]. However, 79% of 411 preoperative US-American patients disagree that anxiety generated by discussion of risks inherent to medical interventions outweighs the benefit of informed consent [3]. Despite these controversies, informed consent is considered as the basis for any treatment in modern medicine [5, 6]. Shared decision-making is not only relevant for surgical procedures, but also for oncological treatments [7]. Oncological patients, in particular, have a high demand for specific information, especially regarding side effects, treatment options, and efficacy, as evidenced by a European survey in 1830 ovarian cancer patients [8]. Identifying possible barriers to shared decision-making and finding solutions to overcome them is therein highly important for improving patient comprehension, which is essential for ensuring the safety of the patient and the success of the treatment [9, 10].

Unfortunately, comprehension rates following informed consent discussions are reported to be alarmingly low. A German pilot project revealed that 45% of patients could not recall even one of the various potential complications discussed prior to a specific operation [11]. Low education levels, language skills, intercultural communication barriers, and short duration of informed consent discussions were found to correlate with lower levels of comprehension [12, 13].

Meeting the quality standards in modern patient-centered shared decision making confronts physicians with many challenges, particularly regarding time and energy constraints inherent to contemporary high-pressure hospital settings [15–17]. Especially in the field of gynecology and gynecological oncology which includes life-changing and emotional situations such as cesareans, chemotherapy, or extensive cancer surgeries, it is essential to conduct the informed consent process thoroughly and empathetically. The goal of the present study was to assess real-world practices and challenges around informed consent in gynecology and gynecological oncology in different types of hospitals across Germany, Switzerland, and Austria.

## Methods

We conducted a multinational, multicentric, anonymous online survey on informed consent practices in gynecological surgery and gynecological oncology medical treatments from February 24, 2022, to September 14, 2022, in Germany, Austria, and Switzerland. The study protocol was approved by the ethics committee of Charité-Universitätsmedizin Berlin (EA1/329/20). The study was registered with the German registry for clinical studies [Deutsches Register klinischer Studien (DRKS)] and received the study registration number DRKS00028295.

The survey was developed as part of a scientific and clinical fellowship program of the JAGO—the Young Academy of Gynecologic Oncology (“Junge Akademie Gynäkologische Onkologie”—JAGO) of the Northeastern German Society of Gynecologic Oncology (“Die Nord-Ostdeutsche Gesellschaft für Gynäkologische Onkologie” – NOGGO e.V.). An online multiple-choice questionnaire was developed under advice and with the input of interprofessional and interdisciplinary experts comprising a total of 64 questions covering informed consent practices in gynecological surgeries and gynecological oncological drug-based tumor therapies.

The questionnaire was validated three times in a pilot group of 21 physicians working in the field of gynecological oncology in regard to clarity of questions and their ability to reach the study´s goals, as well as length and average answering time. The full original German questionnaire and its English translation can be found in the supplementary files.

For categorical variables, multiple answers were allowed when considered necessary. The link to the online questionnaire on the well-evaluated provider SurveyMonkey.com was distributed to hospitals in Germany, Switzerland, and Austria via institutional mailing lists, such as those of the NOGGO and personal contacts. Two reminders were sent. Multiple participations per person were avoided by the SurveyMonkey security system using individualized links, Cookie placements, and IP Address Tracking. Gynecological departments in university and non-university hospitals were invited to participate. Data extractions were performed using Survey monkey. Missing answers were included using the term ‘unanswered’.

Associations between nominally scaled independent variables were analyzed using the chi^2^-test. Normally distributed data were tested for significance using a two-sided Student’s t-test. All statistical tests were carried out with JMP 16 software 22 (SAS^®^). Significance level was set at *p* = 0.05.

## Results

A total of 218 physicians specializing in gynecology answered the survey. Participants of the survey, who reported not to have conducted informed consent discussions for both oncological operations and systemic therapy (*n* = 13), were excluded from the subsequent analysis. Basic demographic information of the respondents can be found in Table 1.

**Table 1:** Demographics of the respondents of the survey

Demographics	Respondents	Percentage of respondents (%)
Total	*n* = 205	
Employing institution		
University hospital	129	62.9
Non-University hospital (maximum care)	51	24.9
Non-University hospital (non-maximum care)	25	12.2
Professional function		
Resident/Assistant doctor	119	58.0
Specialist	44	21.5
Senior consultant	37	18.0
Head of department	5	2.5
Professional experience		
< 1 year	13	6.3
1–5 years	58	28.3
5–10 years	90	43.9
10–20 years	28	13.7
> 20 years	14	6.8

197 respondents stated that they conduct informed consent discussions for gyneco-oncological surgery. Of those, 57 (28.9%) conduct an average of such pre-operative discussions once per week or less, 106 (53.8%) 2–5 times per week and 34 (17.3%) more than five times per week. 87 participants (47.2%) stated that patients generally did not have personal contact with their surgeon prior to their operation, whereas only a minority (*n* = 11, 6.0%) stated that such personal contact happened on a very regular basis.

All professional groups stated that informed consent discussions are most frequently conducted by residents (statement range from 76.5% answered by specialist doctors to 98% answered by residents).

86.4% (*n* = 159) of participants conducted preoperative informative discussions regarding operations that they have never performed themselves or participated in. Further 58.9% (*n* = 106) of respondents stated that they had conducted preoperative informative discussions about operations they had never seen in person.

Significant differences between the answering pattern of these items could be found depending on the professional function of the respondents (see Table 2).

**Table 2:** Answer pattern for practices in preoperative informed consent discussion by type of hospital and professional function

	No preoperative meeting with surgeon n (%)	*p*-value (Chi-square test)	Informed consent—never performed operation n (%)	*p*-value (Chi-square test)	Informed consent—never saw operation n (%)	*p*-value (Chi-square test)
Employing institution						
University hospital	57 (49.6)	0.215	105 (90.5)	0.083	74 (64.4)	0.066
Non-University hospital (maximum care)	24 (52.2)		37 (82.2)		24 (54.6)	
Non-University hospital (non-maximum care)	6 (26.1)		17 (73.9)		8 (38.1)	
Professional function						
Resident/Assistant doctor	55 (55.0)	**0.007**	97 (96.0)	** < 0.001**	77 (77.0)	** < 0.001**
Specialist	23 (53.5)		38 (88.4)		22 (52.4)	
Senior Consultant	9 (25.0)		23 (67.7)		7 (21.2)	
Head of Department	0 (0)		1 (20.0)		0 (0)	

154 participants stated that they conduct informed consent discussions for systemic therapy. Of those, 81 physicians (52.6%) declared that they conduct such pre-therapy discussions once per week or less, 61 (39.6%) 2–5 times per week and 12 (7.8%) more than five times per week.

Less than half of the participants declared that they have managed at least one patient under each specific therapy regimen that they discussed with their patients (*n* = 67, 43.5%). Further, 10% of participants declared that over 70% of the therapy regimens for which they conduct informed consent discussions have never been managed by themselves.

No significant differences between the answering pattern of these items could be found depending on the professional function of the participants.

**Fig. 1:**
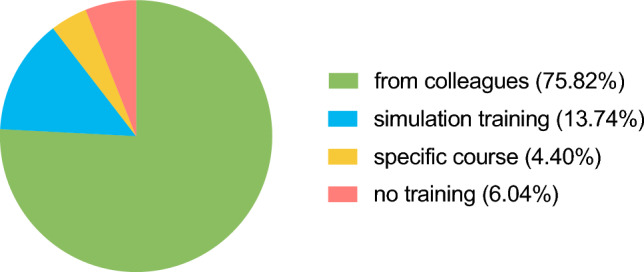
Pie diagram of best teaching method for informed consent

Most participants in the survey stated that they had generally learned how to conduct informed consent discussions with patients from more experienced colleagues (*n* = 138, 76.2%). A smaller portion declared to have additionally been taught in simulation training with or without actor patients (*n* = 24, 13.3%) or during a specific course (*n* = 8, 4.4%), see Fig. 1. Notably, 6.1% (*n* = 11) of participants declared to have never received any form of teaching to that matter. Regarding this aspect, no significant differences were seen between professional groups or type of hospital.

More specifically, 69 (38.1%) participants had never received training specifically on preoperative patient information discussions, while 54 (30%) had never received training specifically on pre-therapeutic patient information about systemic therapy. No significant differences were found between professional groups or type of hospital.

Overall, 172 (94.5%) answered that their hospital does not offer a standardized teaching concept for informed consent discussions with patients. Significant differences were seen in perception of availability of standardized teaching between professional groups: 2 (50.0%) consultants declared that their hospital offers such possibilities, in comparison to a respective 3 (7.5%), and 5 (5.0%) of specialist doctors and residents (*p* = 0.043).

With no significant difference between professional group or type of hospital, 79.1% of participants declared an interest in an online course teaching informed consent discussion, whereas 32.8% of participants declared an interest in such a course that could be attended in person.

161 (85.2%) participants stated that the informed consent discussions they conduct take place in a separate room, offering sufficient privacy for the patient. However, 25 (13.2%) stated that these discussions may also in some cases be conducted in the patient´s room in the presence of other patients. The topological setting did not differ between professional groups or type of hospital.

**Fig. 2:**
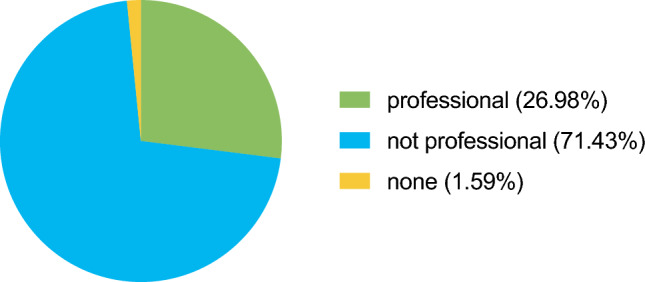
Options for translation for informed consent discussions

When asked about options to overcome language barriers, 51 (27.0%) participants declared having the availability of professional interpreters around the clock, while 3 (1.6%) declared having no possibilities for translation at all. There was a significant difference between type of hospital with 75.6% of university hospital, 51.1% of non-university maximal care hospital, and 91.3% of non-university non-maximal care hospital physicians declaring the possibility of around the clock personal or non-personal professional support for translation (*p* = 0.004). However, most participants (*n* = 135, 71.4%) stated that they had resorted to non-professional options like translation apps, see Fig. 2.

For acquiring signed informed consent, 100 (67.1%) participants declared that their hospital relies solely on analog form (paper). Differences between type of hospital could be found: 33.7% of university hospital, 46.2% of non-university maximal care hospital, and 13.6% of non-university non-maximal care hospital physicians stated that they used digital forms of documentation for signed informed consent (*p* = 0.043).

**Fig. 3:**
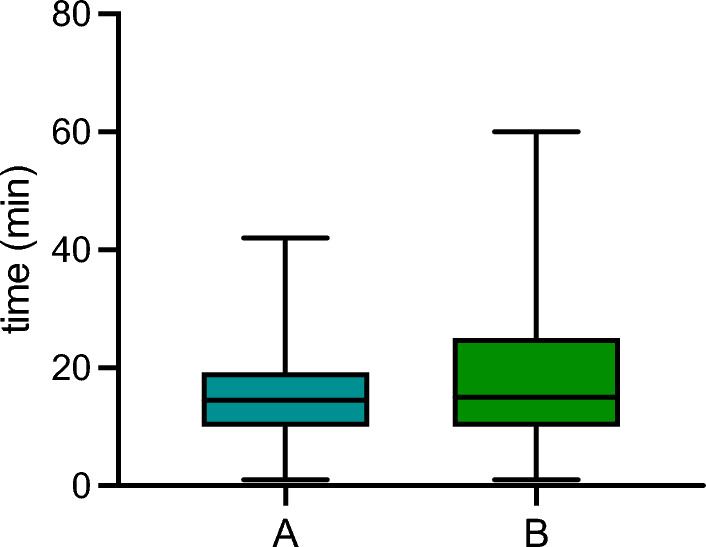
Difference between time estimated to be optimal for informed consent discussion and time available for **A** gyneco-oncological surgery and **B** systemic therapy** in physicians stating not enough time available

Overall, a respective 146 (71.2%) and 132 (64.4%) participants stated that they do not have the time they would deem necessary for informed consent discussions for gyneco-oncological surgery or systemic therapy. Significant differences were seen between professional groups, where 80% (*n* = 4) of consultants vs. 30.3% (*n* = 36) of residents declared having sufficient time for preoperative discussion with their patients (*p* = 0.002) and 80.0% (*n* = 4) of consultants vs. 39.5% (*n* = 47) of residents declared having sufficient time for discussing systemic therapy, respectively (*p* < 0.001). In those reporting insufficient time for informed consent discussions, a mean difference per patient between desired and actual duration of discussion of 14.6 min (Q1–Q3: 10–20 min) for gyneco-oncological surgery (see Fig. 3A) and of 18.2 min (Q1–Q3: 10–25 min) for systemic therapy was declared (see Fig. 3B). These durations did not differ significantly between professional groups and type of hospital. Further 76.1% (*n* = 134) answered that they would not have sufficient time for follow-up questions or discussion with their patients.

Of all the respondents, 9.1% (*n* = 16) declared being generally unsatisfied with the quality of the informed consent discussions they conduct with patients, while 6.3% (*n* = 11) declared being generally very satisfied. These declarations did not differ between professional groups or type of hospital.

**Fig. 4:**
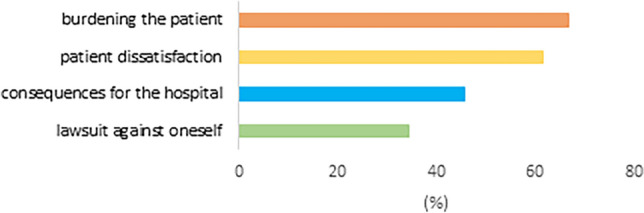
Bar chart of factors leading to psychological burden after informed-consent discussion

Also, 79.7% (*n* = 169) declared feeling a psychological burden or being worried after conducting informed consent discussions. Of those, this feeling was attributed to worrying about a potential lawsuit against oneself in 58 (34.3%), about juristic and/or financial consequences for the hospital in 77 (45.6%), about patient dissatisfaction and criticism in 104 (61.5%) and burdening the patient with too much negative information in 113 cases, respectively (66.9%) (Fig. 4). Significant differences were found regarding professional groups: 7.5% (*n* = 7) of residents vs. 75.0% (*n* = 3) of consultants declared not feeling burdened or worried (*p* = 0.001). Similar differences were also reflected in professional experience, where 12.9% (*n* = 17) of doctors with less than 10 years’ experience vs. 34.3% (*n* = 12) of doctors with more than 10 years’ experience declared no burden or worries (*p* = 0.017).

Over 80% of the participants stated that they have been criticized for an informed consent discussion they conducted by colleagues (*n* = 35, 83.3%), by the patients themselves (*n* = 32, 86.5%) or by relatives of the patients (*n* = 18, 85.7%).

## Discussion

This anonymous survey offers a realistic insight into what is a quintessential part of a doctor’s daily routine. The apparent lack of and desire for structured training to obtain the necessary skills to conduct informed consent discussions is one of the key findings of this study, with almost 80% of participants showing interest in courses teaching such skills.

The demand for a better skills-training does not seem to be unique in gyneco-oncology and is also mirrored in a similar study in orthopedic residents [18]. There, only 33.5% received formal training and many (22.7%) lack confidence in their abilities in obtaining informed consent. 67.1% of all orthopedic residents still feel that they needed more training [18]. Furthermore, there is a large perceptivity gap between heads of department and physicians in training when it comes to the availability of standardized teaching programs in this field, with a discrepancy of 50% to 5% between heads of departments and assistant doctors declaring that teaching programs are available at their hospital. Discrepancies in perception of teaching and setting of doctor–patient discussions may be due to the low number of consultants participating in this survey (*n* = 5), therefore, leading to a potential selection bias. This study revealed another aspect of insufficient teaching in German hospitals, since 77% of assistant doctors answered that they inform patients of operations that they themselves have never seen in an operation theater. Overall, differences of perception and “personal reality” were found between professional groups. Senior respondents had a more positive appreciation of personal interaction in doctor-patient discussions, stating more frequent preoperative personal meetings between patient and surgeons (75% when asking senior doctors vs. 45% assistant doctors).

Preoperative discussion of risks as well as recommendations for patients is acknowledged to play an important role in surgical safety [19, 20]. Procuring informed consent is, therefore, a sensitive subject of great importance to all participants of this study. With rising treatment complexity in gyneco-oncology the challenge to inform our patients sufficiently and individualized increases dramatically. Insecurities, such as fear for patients´ safety, critique or even legal disputes, are strongly connected to the topic. This is expressed by almost 80% of participating physicians, who declared feeling burdened or worried after discussing gyneco-oncologic surgery or systemic therapy with their patients. In most cases, these were attributed to concerns about patient’s safety or dissatisfaction.

One of the main challenges detected in this survey appeared to be the lack of time for qualitative discussions with patients. Most physicians expressed that they would like more time with each patient. The lack of time is a challenge often described in clinical routine in modern medicine [21, 22]. A US-national survey on 579 clinicians in general internal medicine showed that 67% were highly stressed and 62% suffered from high documentation time pressure in a very busy or chaotic workplace (58%) [23]. Such discrepancies between optimal and allotted time for patient contact were also described by primary care doctors in Germany, where differences in perception between more and less experienced physicians could be shown too [24].

Besides sufficient time, further resources for appropriate patient information are required. Especially in a multicultural context, availability of options for overcoming language barriers was low, with overall 27% of participants having professional interpreters available around the clock. These difficulties were previously described in various degrees in other European countries [25, 26]. This shows a potential for optimization in an acknowledged important aspect of modern patient-centered care [27, 28].

It might be considered that the topic of patient information and pretherapeutic discussion could be represented more during medical school. Fortunately, the use of role plays with acting patients in sensitive areas such as “pain management” and “breaking bad news” is becoming increasingly established in medical education. A survey on 1089 physicians and medical students showed that communication training reduces the level of anxiety and reinforces the feeling of self-confidence towards breaking bad news [29]. Therefore, it is possible that a similar concept might be adapted for practicing informed consent discussions for students [30].

To our knowledge, this is the largest multicenter and multinational survey on real-world evidence on informed consent discussions in gyneco-oncology. Since health care and educational systems differ between countries, these results should be validated on an international level. Also, higher numbers of participants to such surveys could reduce biases attributed to relatively higher participation from younger doctors.

The present study is intended to draw attention to the need for providing sufficient resources to medical professionals for appropriate patient informed consent in daily clinical practice. Since this was a subjective survey, further assessments with objective assessment tools are needed to validate our findings.

## Conclusion

This relatively large multinational survey on real world practice of patient informed consent revealed a considerable potential for optimization. Disparities between types of hospital regarding availability of teaching, time for discussion as well as adapting to patient cultural and language background were shown.

## Supplementary Information

Below is the link to the electronic supplementary material.

Supplementary file1 (PDF 110 kb)

Supplementary file2 (PDF 102 kb)

## Data Availability

The datasets used and/or analyzed during the current study are available from the corresponding author upon reasonable request.
