# Supplementary material for: Informed consent in gynecological oncology: a JAGO/NOGGO survey on real-world practices in daily clinical routine
Source: Arch Gynecol Obstet. 2024 Nov 1;311(2):451–7. doi: 10.1007/s00404-024-07776-9 (PMC11890393; doi:10.1007/s00404-024-07776-9)
Supplement: Supplementary file 1 — Supplementary file1 (PDF 110 kb) [file 404_2024_7776_MOESM1_ESM.pdf]

Fragebogen zum Thema: *Ärztliche Aufklärung* (ein Projekt der JAGO e.V.)

1. In welcher Funktion arbeiten Sie im Krankenhaus?

- ☐ Assistenzarzt/ärztin
- ☐ Facharzt/ärztin
- ☐ Oberarzt/ärztin
- ☐ Chefarzt/ärztin

2. Wo arbeiten Sie?

- ☐ Uniklinik
- ☐ Maximalversorgungsklinik
- ☐ kleineres Krankenhaus

3. In welchem Bundesland arbeiten Sie?

4. Wie lange arbeiten Sie schon als Arzt/Ärztin (in Jahren)?

Zeitangabe: ……………………….

5. Welches Geschlecht haben Sie?

- ☐ weiblich
- ☐ männlich
- ☐ divers

6. In welcher Abteilung arbeiten Sie?

- ☐ Gynäkologie
- ☐ Internistische Onkologie
- ☐ Andere: ……………………….

7a. Wie häufig klären Sie auf: onkologische OPs?

- ☐ 0-3 Mal pro Woche
- ☐ 3-10 Mal pro Woche
- ☐ mehr als 10 Mal pro Woche
- ☐ Ich kläre nicht für solche Operationen auf

7b. Wie häufig klären Sie auf: benigne Gyn-OP?

- ☐ 0-3 Mal pro Woche
- ☐ 3-10 Mal pro Woche
- ☐ mehr als 10 Mal pro Woche
- ☐ Ich kläre nicht für solche Operationen auf

7c. Wie häufig klären Sie auf: geburtshilfliche OP?

- ☐ 0-3 Mal pro Woche
- ☐ 3-10 Mal pro Woche

- ☐ mehr als 10 Mal pro Woche
- ☐ Ich kläre nicht für solche Operationen auf

7d. Wie häufig klären Sie auf: medikamentöse Tumortherapie?

- ☐ 0-3 Mal pro Woche
- ☐ 3-10 Mal pro Woche
- ☐ mehr als 10 Mal pro Woche

8. In welchem Setting findet die Aufklärung am häufigsten statt (Mehrfachnennungen möglich)?

- ☐ in einem separatem Raum
- ☐ im Patientenzimmer in Anwesenheit von Mitpatienten
- ☐ im Patientenzimmer ohne Anwesenheit von Mitpatienten
- ☐ in Anwesenheit von Angehörigen

9a. Welche Möglichkeiten stehen Ihnen zur Verfügung, sich der Sprache der Patientin anzupassen (Mehrfachnennungen möglich)?

- ☐ keine, lediglich in deutscher Sprache
- ☐ Übersetzung durch Angehörige
- ☐ Übersetzung durch Klinikpersonal
- ☐ Professioneller Dolmetscher in der Klink
- ☐ Online Übersetzungsprogramme (z.B. „google translate“)
- ☐ Professioneller Dolmetscher per Videotelefonat

9b. Welche der genannten Möglichkeiten steht Ihnen in der Regel rund um die Uhr zur Verfügung (Mehrfachnennungen möglich)?

- ☐ keine, lediglich in deutscher Sprache
- ☐ Übersetzung durch Angehörige
- ☐ Übersetzung durch Klinikpersonal
- ☐ Professioneller Dolmetscher vor Ort
- ☐ Professioneller Dolmetscher per Videotelefonat

10a. Welche Materialien verwenden Sie zur Aufklärung (Mehrfachnennung möglich)?

- ☐ Standardisierte Bögen
- ☐ selbst zeichnen
- ☐ selbst demonstrieren
- ☐ Filme
- ☐ Modelle
- ☐ Internet
- ☐ Broschüren/Informationsblätter

10b. Würden Sie sich mehr verfügbare Informationsbroschüren zur Aushändigung an die Patientinnen wünschen?

- ☐ Ja

- ☐ Nein, es gibt genug
- ☐ Nein, kein Interesse auf Seiten der Patienten

11a. Benutzen Sie schriftlich im Freitextbereich des Aufklärungsbogen (Mehrfachnennung möglich):

- ☐ Abkürzungen
- ☐ Fachbegriffe
- ☐ nur deutsche Sprache
- ☐ Ich verwende den Freitextbereich gar nicht, sondern markiere wichtige Passagen in vorgegebenen Text

11b. In welcher Form wird die Aufklärung dokumentiert:

- ☐ In Papierform
- ☐ ausschließlich elektronisch
- ☐ abwechselnd in Papierform oder elektronisch
- ☐ immer beides

12. In wieviel Prozent der Fälle liegen Ihrer Schätzung nach zwischen der Aufklärung und der Operation / dem Beginn der Chemotherapie?

- mehr als 4 Wochen: ………………………
- mehr als 1 Woche: ....
- weniger als 24h: …………………………….

13a. Wer führt in Ihrer Klinik die Aufklärungen durch? (Mehrfachnennung möglich)

- ☐ Chefarzt/*ärztin
- ☐ Oberarzt/*ärztin
- ☐ Facharzt/*ärztin
- ☐ Assistenzarzt/*ärztin
- ☐ PJ

13b. Wer klärt am häufigsten auf?

- ☐ Chefarzt/*ärztin
- ☐ Oberarzt/*ärztin
- ☐ Facharzt/*ärztin
- ☐ Assistenzarzt/*ärztin
- ☐ PJ

13c. Wer klärt am seltensten auf?

- ☐ Chefarzt/*ärztin
- ☐ Oberarzt/*ärztin
- ☐ Facharzt/*ärztin
- ☐ Assistenzarzt/*ärztin
- ☐ PJ

14a. Klärt in Ihrer Klinik der Operateur für seine eigenen Eingriffe auf?

- ☐ Selten
- ☐ Meistens
- ☐ Immer
- ☐ Nicht zutreffend

14b. Gibt es präoperativ einen Kontakt / ein Gespräch zwischen Operateur und Patientin?

- ☐ Selten
- ☐ Meistens
- ☐ Immer
- ☐ Nicht zutreffend

15a. Klären Sie auch über Operationen/Eingriffe auf, die Sie nie durchgeführt haben?

- ☐ Ja
- ☐ Nein
- ☐ Nicht zutreffend

15b. Klären Sie auch über Operationen/Eingriffe auf, die Sie nie gesehen haben?

- ☐ Ja
- ☐ Nein
- ☐ Nicht zutreffend

15c. Wieviel Prozent der Eingriffe/Therapiemaßnahmen, die sie aufklären, haben Sie noch nie gesehen/ betreut? (freie Angabe in Prozent)

- Onkologische Operation:
- ☐ …….. %
- ☐ Nicht zutreffend

- Benigne Operation:
- ☐ …….. %
- ☐ Nicht zutreffend

- Geburtshilfliche OP:
- ☐ …….. %
- ☐ Nicht zutreffend

- Medikamentöse Tumortherapie:
- ☐ …….. %

15d. Klären Sie für andere Fachabteilungen auf (z.B. Magenspiegelung, Ureterstenteinlage)?

- ☐ Ja

- ☐ Nein

16a. Wie haben Sie das Aufklären gelernt? (Mehrfachnennungen möglich)

- ☐ im Studium in der Vorlesung/Kurs
- ☐ im Studium durch Üben unter StudentInnen
- ☐ im Studium mit SimulationspatientInnen
- ☐ Beiwohnen bei Aufklärung durch erfahrene Ärzte
- ☐ gar nicht

16b. Wurden Sie zum Thema operative Aufklärung am Arbeitsplatz geschult?

- ☐ Ja
- ☐ Nein
- ☐ Kaum
- ☐ Nicht zutreffend

16c. Wurden Sie zum Thema medikamentöse Tumortherapie am Arbeitsplatz geschult?

- ☐ Ja
- ☐ Nein
- ☐ Kaum

16d. Wurden Sie zum Thema Aufklärungsfehler am Arbeitsplatz geschult?

- ☐ Ja
- ☐ Nein
- ☐ Kaum

16e. Hat Ihre Klinik ein standardisiertes Konzept zum Teaching/Erlernen der ärztlichen Aufklärung?

- ☐ Ja
- ☐ Nein
- ☐ Kaum

16f. Von wem sind Sie in Hinsicht auf ärztliche Aufklärung (damals) eingearbeitet worden?

- ☐ Chefarzt/ärztin
- ☐ Oberarzt/ärztin
- ☐ Facharzt
- ☐ Assistenzarzt im 3.-5. Weiterbildungsjahr
- ☐ Assistenzarzt im 1. und 2. Weiterbildungsjahr
- ☐ ich wurde von niemandem eingearbeitet

17a. Wie viel Zeit wünschen Sie sich idealerweise für das Aufklärungsgespräch in Minuten?

- Onkologische Gyn-Operation:
  - ☐ …….. min
  - ☐ Nicht zutreffend

- Benigne Gyn-Operation:
- ☐ …….. min
- ☐ Nicht zutreffend

- Geburtshilfliche Operation:
- ☐ …….. min
- ☐ Nicht zutreffend

- Medikamentöse Tumortherapie:
- ☐ …….. min

17b. Wieviel Zeit haben Sie in Wirklichkeit zur Verfügung?

- Onkologische Gyn-Operation:
- ☐ …….. min
- ☐ Nicht zutreffend

- Benigne Gyn-Operation:
- ☐ …….. min
- ☐ Nicht zutreffend

- Geburtshilfliche OP:
- ☐ …….. min
- ☐ Nicht zutreffend

- Medikamentöse Tumortherapie:
- ☐ …….. min

18. Welche Ursachen gibt es Ihrer Meinung nach, dass bei den PatientInnen nach dem Aufklärungsgespräch häufig weniger „hängen bleibt“ als man es sich wünschen würde? (Mehrfachnennungen möglich)

- ☐ Sprachbarriere
- ☐ Kulturelle Unterschiede
- ☐ Niveauunterschiede/zu geringe medizinische Vorbildung
- ☐ Desinteresse
- ☐ Verdrängung/Angst
- ☐ Recht auf Nicht-Wissen
- ☐ Zu wenig Zeit, auf die Fragen/Sorgen der PatientInnen einzugehen

19a. Bieten Sie der Patientin eine Möglichkeit (an), nach dem Gespräch erneut über die Aufklärung zu sprechen?

- ☐ Selten

- Meistens
- Immer

19b. Hätten Sie realistisch Zeit dafür?

- Ja
- Nein

20a. Kennen Sie bei bzw. nach der Aufklärung folgende Empfindungen/Sorgen (Mehrfachnennungen möglich)?

- Keine Sorgen
- Angst vor Fehler mit gesundheitlichen Folgen für die Patientin
- Angst vor Klage
- Angst vor Fehlern mit organisatorischem/juristischem/finanziellem Schaden für die Klinik
- Zeitdruck
- Angst vor postoperativer Unzufriedenheit und Vorwürfen der PatientInnnen
- Sorge, den PatientInnen durch die Aufzählung der Risiken und Komplikationen psychisch zu belasten

20b. Fühlen Sie sich selber bei bzw. nach Aufklärungsgesprächen verunsichert/belastet? (0=gar nicht, 10=maximal)

0 1 2 3 4 5 6 7 8 9 10

21. Wie zufrieden sind sie selber mit der Qualität Ihrer Aufklärungsgespräche?

- Sehr zufrieden
- meistens zufrieden
- relativ zufrieden
- mäßig zufrieden
- unzufrieden

22a. Wurden Sie schon einmal kritisiert für Ihr Aufklärungsgespräch?

- Ja
- Nein

22b. Wenn ja, von wem? (Mehrfachnennungen möglich)

- Kollegen der eigener Klinik
- Kollegen anderer Fachrichtung
- Patientin
- Angehörige

23. Mussten Sie oder Ihre Klink bereits eine Klage bestreiten, bei der die Aufklärung ein Thema war?

- ☐ Ja
- ☐ Nein

24a. Welche der folgenden spezifischen Risiken/Konsequenzen sind Ihrer Meinung nach aus juristischer Sicht die drei wichtigsten, die zu wenig thematisiert werden im Standardaufklärungsbogen für eine Längslaparotomie Ovarialkarzinom-Debulking-OP (Mehrfachnennungen möglich)?

- Blutung mit Transfusionsbedarf
- (Langzeit-)Intensivpflichtigkeit
- Reanimationspflichtigkeit
- Allergischer Schock
- Resektion im Magen-Darm-Trakt mit Stoma und Inkontinenz
- Resektion im Urogenitaltrakt mit Stoma und Inkontinenz
- Schwere Wechseljahresbeschwerden incl. Osteoporose
- Infertilität
- Ileus, auch als Langzeitfolge
- Sepsis, Peritonitis
- Chronische Schmerzen
- Rezidiv
- Intra-/postoperativer Tod
- Kurzdarmsyndrom incl. Mangelernährung
- Veränderung der Stuhlkontinenz
- kognitive Veränderungen nach der Narkose
- Lagerungsschäden
- Blasenatonie
- Fisteln
- verminderte Lebensqualität
- Veränderungen der Sexualität
- Kann ich nicht beurteilen

24b. Welche der folgenden spezifischen Risiken/Konsequenzen sind Ihrer Meinung nach aus PatientInnensicht die drei wichtigsten, die zu wenig thematisiert werden im Standardaufklärungsbogen für eine Längslaparotomie Ovarialkarzinom-Debulking-OP (Mehrfachnennungen möglich)?

- Blutung mit Transfusionsbedarf
- (Langzeit-)Intensivpflichtigkeit
- Reanimationspflichtigkeit
- Allergischer Schock
- Resektion im Magen-Darm-Trakt mit Stoma und Inkontinenz
- Resektion im Urogenitaltrakt mit Stoma und Inkontinenz
- Schwere Wechseljahresbeschwerden incl. Osteoporose
- Infertilität
- Ileus, auch als Langzeitfolge
- Sepsis, Peritonitis

- Chronische Schmerzen
- Rezidiv
- Intra-/postoperativer Tod
- Kurzdarmsyndrom incl. Mangelernährung
- Veränderung der Stuhlkontinenz
- kognitive Veränderungen nach der Narkose
- Lagerungsschäden
- Blasenatonie
- Fisteln
- verminderte Lebensqualität
- Veränderungen der Sexualität
- Kann ich nicht beurteilen

24c. Würden Sie sich für eine Längslaparotomie Ovarialkarzinom-Debulking-OP einen spezifischeren Aufklärungsbogen wünschen, der oben genanntes beinhaltet?

- ☐ Ja
- ☐ Nein
- ☐ Kann ich nicht beurteilen

24d. Fühlen Sie sich ausreichend geschult/vorbereitet, bei Patientinnen mit (fortgeschrittenem?) Ovarialkarzinom Nutzen und Risiken des Eingriffs inklusive oben genannter Aspekte zu besprechen?

- ☐ Ja
- ☐ Nein
- ☐ Nicht zutreffend

24e Welche der folgenden spezifischen Risiken/Konsequenzen sind Ihrer Meinung nach aus juristischer Sicht die drei wichtigsten, die zu wenig thematisiert werden im Standardaufklärungsbogen für medikamentöse Tumortherapie (Mehrfachnennungen möglich)?

- Haarausfall (ggf. irreversibel)
- Fatigue
- Nagelbrüchigkeit
- Hitzewallungen
- Appetitsteigerung / Appetitminderung
- Allergische Reaktion
- Paravasat
- Progress/Rezidiv trotz medikamentöser Tumortherapie
- Kardiotoxizität
- Polyneuropathie (irreversibel)
- Nierenschäden
- Geschmacksveränderungen
- Chemo-Brain (kognitive Beeinträchtigung)
- Hormonumstellungen / Wechseljahresbeschwerden

24f. Welche der folgenden spezifischen Risiken/Konsequenzen sind Ihrer Meinung aus PatientInnensicht die drei wichtigsten, die zu wenig thematisiert werden im Standardaufklärungsbogen für medikamentöse Tumortherapie (Mehrfachnennungen möglich)?

- Haarausfall (ggf. irreversibel)
- Fatigue
- Nagelbrüchigkeit
- Hitzewallungen
- Appetitsteigerung / Appetitminderung
- Allergische Reaktion
- Paravasat
- Progress/Rezidiv trotz medikamentöser Tumortherapie
- Kardiotoxizität
- Polyneuropathie (irreversibel)
- Nierenschäden
- Geschmacksveränderungen
- Chemo-Brain (kognitive Beeinträchtigung)
- Hormonumstellungen / Wechseljahresbeschwerden

25a. Hätten Sie Interesse an einem Online-Schulungsmodul zur Aufklärung?

- ☐ Ja
- ☐ Nein

25b. Welche spezifischen Thematiken würden Sie sich für ein entsprechendes Online-Schulungsmodul wünschen (Mehrfachnennungen möglich)?

- Aufklärung bei kritischen OP-Risiken und Folgen
- Aufklärung kritischer Aspekte der medikamentösen Tumortherapie
- Aufklärung über spezifische Chemotherapie Nebenwirkungen bei häufig in der Gynäkologie verwendeten Substanzen
- Aufklärung und Prophylaxe von Polyneuropathie
- Aufklärung unter Berücksichtigung kultureller und/oder religiöser Aspekte
- Aufklärungen bei Anwesenheit von Angehörigen
- Effizientes und korrektes Aufklären auch unter Zeitdruck
- Notfallaufklärung
- Rechtliche Grundlagen des Aufklärens

25c. In welcher Form könnten Sie sich vorstellen, an einem entsprechenden Online-Schulungsmodul teilzunehmen?

- ☐ Einmalig
- ☐ Wöchentlich über einen einmonatigen Zeitraum
- ☐ Vierteljährlich über einen Zeitraum von einem Jahr

25d. Hätten Sie Interesse an Präsenz-Schulungen zum Thema Aufklärung mit SimulationspatientInnen (= SchauspielpatientInnen)?

- ☐ Ja
- ☐ Nein

Vielen Dank für Ihre Mitarbeit!
