# Supplementary material for: Informed consent in gynecological oncology: a JAGO/NOGGO survey on real-world practices in daily clinical routine
Source: Arch Gynecol Obstet. 2024 Nov 1;311(2):451–7. doi: 10.1007/s00404-024-07776-9 (PMC11890393; doi:10.1007/s00404-024-07776-9)
Supplement: Supplementary file 2 — Supplementary file2 (PDF 102 kb) [file 404_2024_7776_MOESM2_ESM.pdf]

Questionnaire on Informed consent (a project of JAGO e.V.)

1. What is your role in the hospital?
    - o Intern/Assistant Doctor
    - o Resident
    - o Specialist Doctor
    - o Consultant
2. Where do you work?
    - o University Hospital
    - o Maximum Care Hospital
    - o Smaller Hospital
3. In which state do you work?
4. How long have you been working as a doctor (in years)?
    - o Time: ……………………….
5. What is your gender?
    - o Female
    - o Male
    - o Other
6. Which department do you work in?
    - o Gynecology
    - o Internal Oncology
    - o Other: ……………………….

7a. How often do you conduct informed consent discussions for oncological surgeries?

- ☐ 0-3 times per week
- ☐ 3-10 times per week
- ☐ More than 10 times per week
- ☐ I do not conduct informed consent discussions for such surgeries

7b. How often do you conduct informed consent discussions for benign gynecological surgeries?

- ☐ 0-3 times per week
- ☐ 3-10 times per week
- ☐ More than 10 times per week
- ☐ I do not conduct informed consent discussions for such surgeries

7c. How often do you conduct informed consent discussions for obstetric surgeries?

- ☐ 0-3 times per week
- ☐ 3-10 times per week
- ☐ More than 10 times per week
- ☐ I do not conduct informed consent discussions for such surgeries

7d. How often do you conduct informed consent discussions for medication-based tumor therapy?

- ☐ 0-3 times per week
- ☐ 3-10 times per week
- ☐ More than 10 times per week

8. In what setting does the informed consent discussion usually take place (multiple answers possible)?

    - o In a separate room
    - o In the patient's room in the presence of other patients
    - o In the patient's room without other patients present
    - o In the presence of family members

9a. What options do you have to adapt to the patient's language (multiple answers possible)?

- ☐ None, only in German
- ☐ Translation by family members
- ☐ Translation by hospital staff
- ☐ Professional interpreter in the hospital
- ☐ Online translation programs (e.g., "Google Translate")
- ☐ Professional interpreter via video call

9b. Which of these options are available to you 24/7 (multiple answers possible)?

- ☐ None, only in German
- ☐ Translation by family members
- ☐ Translation by hospital staff
- ☐ Professional interpreter on-site
- ☐ Professional interpreter via video call

10a. What materials do you use for informed consent discussions (multiple answers possible)?

- ☐ Standardized forms
- ☐ Self-drawn illustrations

- ☐ Demonstrations
- ☐ Videos
- ☐ Models
- ☐ Internet
- ☐ Brochures/Information sheets

10b. Would you like to have more available brochures to hand out to patients?

- ☐ Yes
- ☐ No, there are enough
- ☐ No, there is no interest from patients

11a. In the free-text section of the informed consent form, do you use (multiple answers possible):

- ☐ Abbreviations
- ☐ Technical terms
- ☐ Only German
- ☐ I do not use the free-text section but highlight important sections in the provided text

11b. How is the informed consent discussion documented?

- ☐ On paper
- ☐ Exclusively electronically
- ☐ Alternately on paper or electronically
- ☐ Always both

12. What do you estimate is the percentage of all cases, that fullfill the following time frames between the informed consent discussion and the operation/start of chemotherapy?

- ☐ More than 4 weeks: ……………………..
- ☐ More than 1 week: ....
- ☐ Less than 24 hours: …………………………..

13a. Who conducts the informed consent discussions in your clinic? (multiple answers possible)

- ☐ Consultant
- ☐ Specialist Doctor
- ☐ Resident
- ☐ Intern/Assistant Doctor
- ☐ Medical student

13b. Who conducts the informed consent discussions most often?

- ☐ Consultant
- ☐ Specialist Doctor
- ☐ Resident
- ☐ Intern/Assistant Doctor
- ☐ Medical student

13c. Who conducts the informed consent discussions least often?

- ☐ Consultant
- ☐ Specialist Doctor
- ☐ Resident
- ☐ Intern/Assistant Doctor
- ☐ Medical student

14a. Does the surgeon in your clinic conduct informed consent discussions for their own procedures?

- ☐ Rarely
- ☐ Mostly
- ☐ Always
- ☐ Not applicable

14b. Is there a preoperative contact/conversation between the surgeon and the patient?

- ☐ Rarely
- ☐ Mostly
- ☐ Always
- ☐ Not applicable

15a. Do you conduct informed consent discussions for surgeries/procedures that you have never performed?

- ☐ Yes
- ☐ No
- ☐ Not applicable

15b. Do you conduct informed consent discussions for surgeries/procedures that you have never seen?

- ☐ Yes
- ☐ No
- ☐ Not applicable

15c. What percentage of the procedures/therapies you conduct informed consent discussions for have you never seen or supervised? (provide percentage)

- ☐ Oncological surgery:
  - ○ …….. %
  - ○ Not applicable
- ☐ Benign surgery:
  - ○ …….. %
  - ○ Not applicable
- ☐ Obstetric surgery:
  - ○ …….. %
  - ○ Not applicable
- ☐ Medication-based tumor therapy:
  - ○ …….. %

15d. Do you conduct informed consent discussions for other departments (e.g. gastroscopy, ureter stenting)?

- ☐ Yes
- ☐ No

16a. How did you learn to conduct informed consent discussions? (multiple answers possible)

- ☐ In lectures/courses during medical school
- ☐ Practicing with fellow students during medical school
- ☐ With simulation patients during medical school
- ☐ Observing informed consent discussions conducted by experienced doctors
- ☐ Not at all

16b. Were you trained on informed consent discussions at your workplace?

- ☐ Yes
- ☐ No
- ☐ Hardly
- ☐ Not applicable

16c. Were you trained on informed consent discussions for tumor therapy at your workplace?

- ☐ Yes
- ☐ No
- ☐ Hardly

16d. Were you trained on possible errors in informed consent discussions at your workplace?

- Yes
- No
- Hardly

16e. Does your clinic have a standardized concept for teaching/learning informed consent discussions?

- Yes
- No
- Hardly

16f. Who trained you in informed consent discussions (at that time)?

- Chief Physician
- Senior Doctor
- Specialist Doctor
- Assistant Doctor in the 3rd-5th year of training
- Assistant Doctor in the 1st and 2nd year of training
- I was not trained by anyone

17a. How much time would you ideally like for an informed consent discussion (in minutes)?

- Oncological gynecological surgery:
  - …….. min
  - Not applicable
- Benign gynecological surgery:
  - …….. min
  - Not applicable
- Obstetric surgery:
  - …….. min
  - Not applicable
- Medication-based tumor therapy:
  - …….. min

17b. How much time do you really have?

- Oncological gynecological surgery:
  - …….. min

- Not applicable

- Benign gynecological surgery:
  - …….. min
  - Not applicable
- Obstetric surgery:
  - …….. min
  - Not applicable
- Medication-based tumor therapy:
  - …….. min

18. In your opinion, what are the reasons that patients often remember less after the informed consent discussion than desired? (multiple answers possible)

- Language barrier
- Cultural differences
- Differences in education/too little medical background
- Lack of interest
- Suppression/Fear
- Right not to know
- Too little time to address the patient's questions/concerns

19a. Do you offer the patient an opportunity to talk to you again about the informed consent discussion afterwards?

- Rarely
- Mostly
- Always

19b. Do you realistically have time for it?

- Yes
- No

20a. During or after the informed consent discussion, do you experience the following feelings/concerns? (multiple answers possible)

- No worries
- Lack of time
- Lack of knowledge
- Fear of possible mistakes

- ☐ Insufficient training
- ☐ Little interest from the patient
- ☐ Anger
- ☐ Uncertainty due to a language barrier
- ☐ Lack of specialist vocabulary in another language
- ☐ Not always having the opportunity to conduct the conversation again

20b. Do you feel uncertain/burdened during or after informed consent discussions? (0=not at all, 10=maximally)

____

0 1 2 3 4 5 6 7 8 9 10

21. How satisfied are you with the quality of your informed consent discussions?

- ☐ Very satisfied
- ☐ Mostly satisfied
- ☐ Relatively satisfied
- ☐ Moderately satisfied
- ☐ Dissatisfied

22a. Have you ever been criticized for your informed consent discussion?

- ☐ Yes
- ☐ No

22b. If yes, by whom? (Multiple answers possible)

- ☐ Colleagues from your clinic
- ☐ Colleagues from other specialties
- ☐ Patient
- ☐ Relatives

23. Have you or your clinic ever faced a lawsuit where informed consent was an issue?

- ☐ Yes
- ☐ No

24a. Which of the following specific risks/consequences do you think are the three most important from a legal perspective that are underrepresented in the standard informed consent form for a longitudinal laparotomy ovarian cancer debulking surgery? (Multiple answers possible)

- ☐ Bleeding requiring transfusion

- ☐ (Long-term) intensive care requirement
- ☐ Requirement for resuscitation
- ☐ Allergic shock
- ☐ Resection in the gastrointestinal tract with stoma and incontinence
- ☐ Resection in the urogenital tract with stoma and incontinence
- ☐ Severe menopausal symptoms including osteoporosis
- ☐ Infertility
- ☐ Ileus, also as a long-term consequence
- ☐ Sepsis, peritonitis
- ☐ Chronic pain
- ☐ Recurrence
- ☐ Intra-/postoperative death
- ☐ Short bowel syndrome including malnutrition
- ☐ Changes in stool continence
- ☐ Cognitive changes after anesthesia
- ☐ Intraoperative positioning nerve injuries
- ☐ Bladder atony
- ☐ Fistulas
- ☐ Reduced quality of life
- ☐ Changes in sexuality
- ☐ Cannot assess

24b. Which of the following specific risks/consequences do you think are the three most important from a patient's perspective that are underrepresented in the standard informed consent form for a longitudinal laparotomy ovarian cancer debulking surgery? (Multiple answers possible)

- ☐ Bleeding requiring transfusion
- ☐ (Long-term) intensive care requirement
- ☐ Requirement for resuscitation
- ☐ Allergic shock
- ☐ Resection in the gastrointestinal tract with stoma and incontinence
- ☐ Resection in the urogenital tract with stoma and incontinence
- ☐ Severe menopausal symptoms including osteoporosis
- ☐ Infertility

- Ileus, also as a long-term consequence
- Sepsis, peritonitis
- Chronic pain
- Recurrence
- Intra-/postoperative death
- Short bowel syndrome including malnutrition
- Changes in stool continence
- Cognitive changes after anesthesia
- Intraoperative positioning nerve injuries
- Bladder atony
- Fistulas
- Reduced quality of life
- Changes in sexuality
- Cannot assess

24c. Would you prefer a more specific informed consent form for a longitudinal laparotomy ovarian cancer debulking surgery that includes the aforementioned risks?

- Yes
- No
- Cannot assess

24d. Do you feel adequately trained/prepared to discuss the benefits and risks of the procedure, including the aforementioned aspects, with patients with (advanced?) ovarian cancer?

- Yes
- No
- Not applicable

24e. Which of the following specific risks/consequences do you think are the three most important from a legal perspective that are underrepresented in the standard informed consent form for drug-based tumor therapy? (Multiple answers possible)

- Hair loss (potentially irreversible)
- Fatigue
- Brittle nails
- Hot flashes
- Increased/decreased appetite

- Allergic reaction
- Extravasation
- Progression/recurrence despite drug-based tumor therapy
- Cardiotoxicity
- Irreversible polyneuropathy
- Kidney damage
- Taste changes
- Chemo-brain (cognitive impairment)
- Hormonal changes/menopausal symptoms

24f. Which of the following specific risks/consequences do you think are the three most important from a patient's perspective that are underrepresented in the standard informed consent form for drug-based tumor therapy? (Multiple answers possible)

- Hair loss (potentially irreversible)
- Fatigue
- Brittle nails
- Hot flashes
- Increased/decreased appetite
- Allergic reaction
- Extravasation
- Progression/recurrence despite drug-based tumor therapy
- Cardiotoxicity
- Irreversible polyneuropathy
- Kidney damage
- Taste changes
- Chemo-brain (cognitive impairment)
- Hormonal changes/menopausal symptoms

25a. Would you be interested in an online training module on informed consent?

- Yes
- No

25b. Which specific topics would you like to see covered in an online training module? (Multiple answers possible)

- Informed consent for critical surgery risks and consequences

- Informed consent for critical aspects of drug-based tumor therapy
- Informed consent for specific chemotherapy side effects of substances commonly used in gynecology
- Informed consent and prevention of polyneuropathy
- Informed consent considering cultural and/or religious aspects
- Informed consent in the presence of relatives
- Efficient and correct informed consent even under time pressure
- Emergency informed consent
- Legal basics of informed consent

25c. In what format could you imagine participating in an online training module?

- One-time
- Weekly over a one-month period
- Quarterly over a one-year period

25d. Would you be interested in face-to-face training on informed consent with simulation patients (= actor patients)?

- Yes
- No

Thank you for your cooperation!
